# Antioxidant, anti-inflammatory, and immunomodulatory pathways of *Citrus limon* juice in a respiratory-irritated rat model.

**DOI:** 10.3389/fnut.2026.1743575

**Published:** 2026-04-22

**Authors:** Reham M. Algheshairy, Hoda A. Ali

**Affiliations:** 1 Department of Food Science and Human Nutrition, College of Agriculture and Food, Qassim University, Buraydah, Saudi Arabia; 2 Department of Nutrition and Clinical Nutrition, College of Veterinary Medicine, Cairo University, Cairo, Egypt

**Keywords:** bioactive constituent, glycolic acid, *Citrus limon* juice, inflammatory cytokine, lung antioxidant enzyme, lymphocyte proliferation phagocytic index, antioxidant

## Abstract

**Introduction:**

Respiratory injuries with immunosuppression represent a challenge worldwide. This work aimed to clarify the efficacy of Hesawi *Citrus limon* juice (lemon juice, LJ) in ameliorating respiratory irritation in a model rat induced by surgical intratracheal instillation of glycolic acid.

**Methods:**

Rats were assigned to five groups. Groups (1 and 2): normal and negative control, respectively; Group (3): positive model group; Groups (4 and 5): treated groups that received 3 and 6 mL/kg/day LJ orally, respectively. At 3 weeks of the experiment, groups 2, 3, 4, and 5 underwent surgical intratracheal instillation with 0.2 mL/kg body weight saline for group 2 and 0.2 mL/kg body weight 5 % glycolic acid for groups 3, 4, and 5.

**Results:**

The model group exhibited inflammatory signs, evidenced by a significant increase in the total leucocyte count (*P* < 0.01), neutrophils % (*P* < 0.05), inflammatory cytokines (TNF-α, IL-6), and C-reactive protein (CRP), accompanied by a decrease in lymphocytes % and an increase in neutrophils/lymphocyte ratio. Likewise, model rats exhibited significant adverse alterations (*P* < 0.05) in the phagocytic index and in glucose consumption with lymphocytes stimulated with either lipopolysaccharide or phytohemagglutinin-P. The high dose of LJ significantly (*P* < 0.05) mitigated the inflammatory signs to a greater extent than the low dose. Lung tissue glutathione peroxidase, superoxide dismutase, and catalase activities were reduced in model rats, whereas a high LJ dose significantly elevated them (*P* < 0.05). A high dose of LJ supplementation normalized TNF-α, IL-6, and CRP levels.

**Discussion:**

Consistent with the other data, histological examination of the lung and trachea showed only slight or no inflammatory lesions, with the effect most pronounced at the high dose. In conclusion, the bioactive constituents of LJ control oxidative stress and inflammation, enhance immune function, and, in a dose-dependent manner, ameliorate lung tissue irritation induced by surgical intratracheal instillation of glycolic acid.

## 1 Introduction

Respiratory disorders represent a risk to public health globally, depending on their prevalence and economic factors. The problem includes both infectious and non-communicable diseases. In 2019, there was an increase in respiratory issues, which are regarded as the third leading cause of death, responsible for 4.0 million deaths, with a prevalence of 454.6 million cases (1). In the Kingdom of Saudi Arabia (KSA), the prevalence and incidence of chronic obstructive pulmonary disease have been steadily rising since 2019 by about 330% compared with the number recorded in 1990 (2). On the other hand, the top 10 causes of death in KSA include lower respiratory infections, which emphasizes the problem.

The lung is usually exposed to many environmental pollutants, including smoke, pollen, sandstorms, and volatile organic compounds, which harm the respiratory and circulatory systems (3). Consequently, many lethal infectious diseases may develop, besides other respiratory dysfunctions (4, 5). Air pollutants have a detrimental effect on the immune system since they can induce oxidative stress, creating free radicals, which may harm the respiratory system and lower immunity, subsequently increasing the susceptibility to infections (6). In 2016, the Global Health Observatory reported that air pollution was responsible for approximately 67% of respiratory disease mortality. Therefore, immunostimulants should receive great attention; one of the many theories relating to the immune response is nutrition (7, 8), along with the concept of the anti-inflammatory component (9) and antioxidant capacity (10).

*Citrus limon* (Lemon) is one of the most abundant citrus crops in the world, Family, Rutaceae. Lemon has various health benefits in both vivo and vitro, including anti-inflammatory, anti-oxidative, immunomodulatory, anticancer effects, antimicrobial effects, hypolipemic effects, and cardiovascular disorders protection (11–13). Vitamins, minerals, and polyphenols (mostly flavonoids) found in high concentrations in lemons include hesperidin, eriocitrin, naringin, neohesperidin, quercetin, chlorogenic acid, luteolin, and kaempferol (12). Citrus fruits are rich in vitamin C, which plays a crucial role in maintaining the integrity of the mucosal immune barriers (6). Vitamin C reduces infection susceptibility, especially in the respiratory tract (14).

Hesawi *Citrus limon* is the most important fruit grown in Al-Hassa, eastern KSA. It is distinguished by its potent aroma and flavor, the delicate peel, and the copious amounts of juice from other citrus fruits. Even days after picking, it still smells and has its original elements, and even after being stored for several months without adding a preservative, the green color does not change (15). Given that fruit juices are susceptible to alteration during extraction and storage (16). It is prudent to investigate the anti-inflammatory, antioxidant, and immunomodulatory properties of Hesawi lemon juice.

Depending on the concentration, glycolic acid is used as a chemical peeling agent (17). According to reports, several corrosive acidic agents, including glycolic acid, can harm the lungs by causing pulmonary edema and alveolitis (18). Glycolic acid damages bronchial and bronchiolar epithelial cells and causes acute hemorrhagic alveolitis and pulmonary edema (19). Increased oxidative stress from glycolic acid could elevate inflammatory cytokines, such as TNF-α and IL-6, in the bronchoalveolar lavage fluid of rats exposed to single and repeated inhalations (20). Humans are oronasal breathers, whereas rodents are obligatory nasal breathers. Because nasal breathing provides more effective filtration of particles and gases than oral breathing, a greater proportion of inhaled material reaches the peripheral airways in humans (20). This makes it difficult to induce lower respiratory tract irritation in rodents via standard inhalation. Therefore, surgical intratracheal instillation of glycolic acid was chosen in this study to cause lung irritation.

Despite the recognized potential of Citrus limon in mitigating inflammatory conditions, a significant gap remains in the literature. The present study employs, for the first time, a surgical intratracheal instillation model of glycolic acid to investigate the protective effects of Hesawi lemon juice against lung irritation. This approach is critical, as the surgical model more accurately replicates the human oronasal breathing pattern, in which the lower respiratory tract is more susceptible to injury. Furthermore, the specific antioxidant, anti-inflammatory, and immunomodulatory pathways through which the bioactive compounds of traditionally stored Hesawi lemon juice (without preservatives) confer protection remain largely unexplored. Therefore, this study was designed to provide a novel and comprehensive evaluation of the efficacy of Hesawi lemon juice in alleviating respiratory irritation induced by surgical intratracheal instillation of glycolic acid. By integrating phytochemical profiling with assessments of antioxidant capacity, anti-inflammatory effects, and immunological integrity, this work seeks to elucidate the underlying protective mechanisms and establish a scientific rationale for its use in respiratory health.

## 2 Materials and methods

### 2.1 Ethical standard

The current work, as reported by the International Animal Ethics Committee, was approved by the Deanship of Scientific Research at Qassim University in the Kingdom of Saudi Arabia’s Committee of Health Research Ethics under the number “22-10-11.” *This study was in accordance with the Animal Research: Reporting In Vivo Experiments (ARRIVE) Essential 10 guidelines.*

### 2.2 Experimental animals

Adult healthy male Wistar rats weighing 160 ± 10 g were obtained from the University of King Saud laboratory center in Riyadh, KSA. The animals were housed in cages in an animal-specific rearing room at the College of Agriculture at Qassim University in Saudi Arabia. The animals were housed under a 12-h light/dark cycle at 23 ± 2°C temperature and50% ± 3% relative humidity. The rats were acclimatized for 1 week and were offered a commercial diet and fresh, clean water *ad libitum*. The commercial diet was obtained from “The Wafi Company for Animal Feed, Qassim, KSA.” The diet is formulated to furnish nutrient requirements for lab animals as recommended by the National Research Council (21). General guidelines for using and caring for animals for scientific purposes were considered. This experiment follows the animal care guidelines suggested by Qassim University’s Deanship of Scientific Research and the “International Animal Ethics Committee.” All procedures involving animals complied with the animal care and ethical standards mandated by the Deanship of Scientific Research at Qassim University.

### 2.3 Hesawi *Citrus limon*

Hesawi *Citrus limon* (LJ) is cultivated in the Al-Hassa province of KSA. According to the traditional procedures followed there, LJ is extracted by squeezing the fruit and stored without chemical preservatives in dark glass bottles with narrow necks. A thin layer of olive oil is poured on top of the bottle to isolate the juice from the air, and the bottle is then tightly sealed. The bottle is first exposed to sunlight for 2 h, with the duration adjusted according to sunlight intensity and weather conditions, then stored in a refrigerator for several months, and finally used for human consumption. This technique mimics the preservation method described by Sindhu and Khatkar (22), though they used heat processing in a hot water bath at 80°C instead of sunlight. The LJ used in this study was purchased from a local market in Al-Hassa, KSA.

### 2.4 High-performance liquid chromatography for bioactive compounds of lemon juice

The process described in Agilent Application Note Publication 5991-3801EN, 2014, was used. Agilent 1260 infinite HPLC Series (Agilent, United States) with a quaternary pump was used with the a Kinetex^®^ 1.7 um EVO C18 50 mm × 2.1 mm column (Phenomenex, United States). It was run at 30°C. A ternary linear elution gradient employing (A) HPLC-grade water with 0.1% H3PO4 (v/v), (B) acetonitrile with 0.1% H3PO4 (v/v), and (C) methanol, flow rate of 0.2 mL/min, is used to achieve separation. The volume injected was 20 μL. Detection: The variable wavelength detector was at 280 nm, a standard wavelength for phenolic compounds and flavonoid detections based on their characteristic UV absorption (23). The temperature and humidity are 20 °C and 38% RH, respectively.

### 2.5 HPLC for the organic acids of lemon juice

To find the organic acids in LJ, an inert solution was used in an HPLC analysis. The separation was performed using the Eclipse AQ-C18 HP column, which is 4.6 mm × 150 mm i.d., 3 m (GL Sciences, Japan). The mobile phase was made up of 0.005 N sulfuric acid. The linear gradient for flow rate was sequentially programmed for the mobile phase as follows: 0.8 mL/min for the first 4.5 min; 1 mL/min for the next 4.7–4.7 min; 1.2 mL/min for the next 4.71–8.8 min; 1.3 mL/min for the next 9.8–23 min; and 0.8 mL/min for the final 23–25 min. The Diode Array Detector (DAD) was monitored at 210 nm, which is optimal for detecting organic acids due to the absorption of the carboxyl moiety. For every one of the samples, a volume of 5 μL was used. The temperature in the column was kept at 55 °C.

### 2.6 Antioxidant activity of lemon juice

An assay method by Brand-Williams (24) was used to determine 2, 2-Diphenyl-1-picrylhydrazyl (DPPH) using UV/Vis. Spectrophotometer (Jenway, England). Methanol was used to prepare concentrations from 2/100 g to 10/100 g from a sample. DPPH radical (100 μL, 0.2mM) and extract (100 μL) were dissolved in methanol. After stirring, the mixture was kept for 15 min in the dark. The absorbance of the DPPH radical solution was then measured at 517 nm, its characteristic absorption maximum. The test was conducted at 25°C with a 38% RH. [(Ao-A1)/Ao] × 100 was used to compute the percentage scavenging effect, where Ao is the absorbance without the sample, and A1 is the absorbance with the sample.

### 2.7 Vitamin C (ascorbic acid) in lemon juice

Lemon juice was prepared for vitamin C determination by the volumetric method (25), using 2, 6-dichlorophenol indophenol dye as an indicator and following the equation:


Amount⁢of⁢vitamin⁢C⁢mg/100⁢mL⁢sample=

$$\frac{0.5\,\mathrm{mg}}{\mathrm{V1}\,\mathrm{ml}}\times \frac{\mathrm{V2}\,\mathrm{ml}}{5\,m\,l}\times \frac{100\,M\,L}{\mathrm{Wt}}\times 100$$

Where the amount of dye consumed from a working standard solution represents V1, the amount of dye consumed from a sample represents V2, and the weight of the sample represents Wt.

### 2.8 Surgical intratracheal instillation of glycolic acid, induction of lower lung irritation (model animal)

Intratracheal instillation was done under diethyl ether anesthesia through inhalation of diethyl ether ≥ 99.0% (Sigma-Aldrich, Germany) at a concentration of 1.9% (0.08 mL per liter of the chamber used) according to Johns Hopkins University Animal Care and Use Committee (ACUC). The calculated amount of ether was presented in a cotton ball inside the animal chamber, but without direct contact with the animal. Although diethyl ether has irritating and flammable disadvantages, all precautions were taken. The trachea was exposed surgically, and then a needle with 5% glycolic acid (Cas no. 79-14-1, Sigma-Aldrich, United States) at a dose of 0.2 mL/kg body weight (19) was inserted between the tracheal rings (26). Surgical intratracheal instillation was chosen in the current study because of the oronasal breathing habits in humans, while rats are compelling nose breathers, making the lower respiratory tracts more susceptible to pulmonary affections in humans (27).

### 2.9 Experimental procedures and design

Forty male Wistar rats were divided into five groups, with eight rats in each. Groups (1, 2, and 3): normal, negative, and positive (a model positive), respectively, received no supplementation during the experiment. Group (4 and 5): treated groups received daily oral gavage with 3 and 6 mL/kg/day LJ (28), respectively, throughout the experimental period. Surgical intratracheal instillation was performed on all groups except Group 1 (normal control) 3 weeks after the experiment commenced. Rats in group 2 were exposed to an intratracheal instillation of 0.2 mL/kg body weight of saline (negative control). Whereas, group 3 (positive), groups 4 and 5 (treated) were exposed to intratracheal instillation with 0.2 mL/kg body weight 5% glycolic acid (19) (Table 1).

**TABLE 1:** Experimental design.

Treatments\Groups	Normal group G1	Negative group G2	Positive group G3	Treated group G4	Treated group G5
lemon juice	–	–	–	3 mL/kg/day	6 mL/kg/day
Three weeks after the beginning of the experiment					
Surgical intratracheal operation	–	√	√	√	√
Intratracheal instillation 0.2 mL/kg BW saline	–	√	–	–	–
Intratracheal instillation 0.2 mL/kg BW 5% glycolic acid	–	–	√	√	√
On days 1 and 7 post-intratracheal installation, samples were collected for measurements					

Comparing G1 and G2 to ensure that the surgical intratracheal operation itself does not affect the results. Comparing G2 and G3 to detect the lung irritation induced by glycolic acid. Comparing G3 vs. G4 and G5 to evaluate the efficacy of lemon juice in the prevention of respiratory irritation.

### 2.10 Clinical observations and body weight

Body weights were recorded at 3 weeks into the experimental period (pre-intratracheal instillation), on day 1 post-intratracheal instillation, and then day after day for 1 week. The clinical signs and symptoms were observed post-intratracheal instillation for 1 week to detect any illness, weakness, or death.

### 2.11 Sample collection and processing

On the first and seventh days (days 1 and 7) post intratracheal instillation, diethyl ether was used to anesthetize rats; they were bled using the ocular method and divided into two portions: one with anticoagulant (for hematological and phagocytic assays) and one without (for serum separation). Whole blood samples from each group were used to determine the total leucocyte count (TLC) and differential leucocyte count. The phagocytic index was calculated using the blood sample with an anticoagulant. Glucose consumption by lymphocytes in the incubation medium of mitogens triggered by either lipopolysaccharide (LPS) or phytohemagglutinin-P (PHA-P) was detected in blood samples collected on day 7 post-surgery. Serum was collected to determine the levels of the inflammatory cytokines, tumor necrosis factor-alpha (TNF-α), interleukin-6 (IL-6), and C-reactive protein (CRP).

Three animals from each group were randomly selected, and euthanasia was performed to reduce the pain. The rats were anesthetized with diethyl ether (as mentioned in surgical intratracheal instillation), as a part of step one of the euthanasia, the animals were first rendered unconscious. Followed by step two, the euthanasia was done physically very quickly by cervical dislocation according to the consulting American Veterinary Medical Association (AVMA) Guidelines for the Euthanasia of Animals, 2020. The trachea (the route of instillation and a site of potential inflammatory response), lungs (the primary site of glycolic acid-induced damage), and spleen (the main organ for immune response) were gently and aseptically removed. Lung tissue was analyzed for antioxidant enzyme activities, and spleen tissue was used to assess lymphocyte viability. Sections of the trachea and lung were preserved for histopathological examination

### 2.12 Hematological parameters (leucogram)

On day 7 post-instillation, total leukocyte count (TLC) was determined from anticoagulated blood samples using a hemocytometer, following the method described by Coles (29). Differential leukocyte counts [neutrophils (N%), lymphocytes (L%), eosinophils %, and monocytes %] were performed on blood smears using the cross-sectional method (30). The neutrophil-to-lymphocyte (N/L) ratio was subsequently calculated.

### 2.13 Phagocytic activity (phagocytic index, phagocytosis)

Animals utilized in the investigation had their phagocytic activity measured using an in vitro carbon clearance technique. Blood samples were used on day 1 and day 7 post-intratracheal instillation of glycolic acid. Blood was drawn from 1.5 mL of heparin (50 IU/mL). After being centrifuged at 3,000 g for 30 min, the blood sample was combined with 6 μl of the supernatant fraction of India ink (Pelikan AG D-3,000, Hanover, Germany). Each sample was combined and divided into three equal aliquots. The aliquots were then incubated at 37 °C, and measurements were taken at 20 and 40 min. After incubation, 150 μL of each blood and India ink mixture was diluted in 2 mL of saline. The samples were then centrifuged at 50 g for 4 min. The optical density of the resulting supernatant was measured at 535 nm using a spectrophotometer, with the background set to zero. As more carbon was phagocytosed over time, ODs decreased. The phagocytic index was detected as the slope of the regression of OD (log2) on time after OD data were scaled to a log2 scale (h) (31).

### 2.14 Glucose consumption assay (lymphocyte function)

With some modifications, the glucose consumption test was used to assess lymphocyte transformation (32). The test goal is to identify whether a body has a T and B cell response against a particular medication. A Ficoll histopaque solution (Sigma^®^ H8889 *d* = 1.077 g mL^–1^) layer was placed atop citrated blood from various groups at the end of the week after being diluted 1:1 with phosphate-buffered saline (PBS, pH 7.2). The mononuclear cell layer was aspirated after the blood was centrifuged at 3,500 rpm for 30 min. PBS was used to wash the cells three times, and the RPMI-1640 medium was applied twice (Gibco Laboratories, Paisley, United Kingdom) complemented with fetal calf serum 10% (Gibco Laboratories, Paisley, United Kingdom), 2 mmol/l l-glutamine (Sigma Laboratories, St. Louis, Mo, United States), 100 IU/mL penicillin and 100 mg/mL streptomycin (Sigma Laboratories, St. Louis, Mo, United States). The trypan blue dye exclusion method was used to analyze the cells after they had been resuspended in the medium. Under a light microscope, the viable (unstained) cells were counted with a hemocytometer. According to Marko et al. (33), LPS (Sigma, United States) was utilized as a B-cell mitogen, and PHA-P (Sigma-Aldrich, United States) was used as a T-cell mitogen (34). In 24-well plates (Corning Costar^®^ No. 3526), lymphocytes were cultivated in triplicate in the presence of either 5 g/mL PHA-P or 10 μg/100 μL LPS. Each well contained 200 μL of culture solution with 2 × 106 cells and was incubated for 72 h. Cultures were kept at 37°C in a humid atmosphere with 5% CO2. The amount of glucose in the final incubation medium was calculated using commercial colorimetric assay kits (Cayman Chemical, United States). The glucose consumption (mg/dL) was lower in the control samples than in the stimulated cell cultures, indicating lymphocyte stimulation.

### 2.15 Splenocyte viability %

Splenocyte viability was assessed using the trypan blue exclusion method. In a nutshell, 20 μL of the homogenate and 20 μL of Trypan blue dye (GIBCO/BRL) were combined. Using an inverted microscope (Olympus, Japan), the total number of unstained and blue-dyed cells (dead cells) was counted using a hemocytometer. The following formula was used to compute the percentage of viable cells as a proxy for cell proliferation.


$$\mathrm{Cellular}\,\mathrm{viability}\%=\frac{\left(\mathrm{No}.\mathrm{of}\,\mathrm{Total}\,\mathrm{cells}-\mathrm{No}.\mathrm{of}\,\mathrm{dead}\,\mathrm{cells}\right)}{\mathrm{No}.\mathrm{of}\,\mathrm{Total}\,\mathrm{cells}}\times 100$$

### 2.16 Lung tissue antioxidant enzyme activities

On day 7 post-instillation, the lungs and trachea were gently removed. The lung was divided into two portions; one portion, along with the trachea, was preserved for histological examination. The other lung portion was cleaned with saline in an ice bath and homogenized with ice-cold Tris-HCl (Sigma-Aldrich, United States) in a ratio of 1:10 (w:v) (0.1M, pH 7.4) (35). The homogenates were kept at −80°C until use. The suspended mixture was centrifuged in a cooled centrifuge at 3,000 rpm for 10 min at 4°C. The resultant supernatant was employed to measure the activity of antioxidant enzymes. Three enzymes were assessed: glutathione peroxidase (GSH-Px), superoxide dismutase (SOD), and catalase (CAT), using kits (Biodiagnostic Company, Cairo, Egypt), Catalogue No. (GP 25 24, SD 25 21, and CA 26 18, respectively). The absorbencies of GSH-Px, SOD, and CAT were spectrophotometrically measured at 340 nm, 560 nm, and 520 nm, respectively.

### 2.17 Circulating inflammatory cytokines (TNF-α, IL-6) and C-reactive protein

Serum levels of the inflammatory cytokine TNF-α were determined using ELISA kits (SunLong Biotech Co., Ltd., China), Cat. No., SL722Ra and IL-6 were performed using ELISA kits (Abcam, Cambridge, MA, United States) Cat. No., ab234570. CRP was performed using ELISA kits (SunLong Biotech Co., Ltd., China), Cat. No, SL0202Ra, the manufacturer’s instructions were followed.

### 2.18 Histopathological observations

Specimens from the trachea and lung were used for histopathological examinations. The tissues were treated using standard paraffin wax after being preserved in a 10% neutral buffered formalin aqueous solution. Hematoxylin and eosin (H&E) stain was used to stain the slices for histological examination under a light microscope (36).

### 2.19 Statistical methods

Data are presented as the mean ± standard error (SE). A one-way analysis of variance (ANOVA) was performed for each measured parameter using SAS software (version 20; SAS Institute, United States), with treatment considered a fixed effect. When ANOVA revealed significant overall differences (*P* < 0.05), Tukey’s Honestly Significant Difference (HSD) *post-hoc* test was applied for pairwise comparisons. Specifically, the negative control group was compared with all other experimental groups, and the positive control (model) group was compared with the treatment groups (Groups 4 and 5). Statistical significance was set at *P* < 0.05 and *P* < 0.01.

The normality of residuals was assessed using the Shapiro–Wilk test, and homogeneity of variances was evaluated using Levene’s test. All datasets met the assumptions of normality and homoscedasticity (*P* > 0.05). Non-parametric alternatives (e.g., Kruskal–Wallis’s test followed by Dunn’s *post-hoc* test) were considered for parameters where assumptions were not fully met; however, because all assumptions were satisfied, the results reported are based on parametric tests.

The following linear model was applied:


$$Y_{i\,j}=\mu +T_{i}+\varepsilon_{i\,j}$$

where Yij is the observed value for the animal j in treatment i, μ is the overall mean, Ti is the fixed effect of treatment i, and εij is the random error term.

## 3 Results

Overall, no significant differences were found in the results for all parameters used in this study between the normal group (without surgical intratracheal operation) and the negative group (with surgical intratracheal operation using saline solution). This suggests that the surgical operation did not affect the results.

### 3.1 The phenolic compounds of lemon juice

**FIGURE 1:**
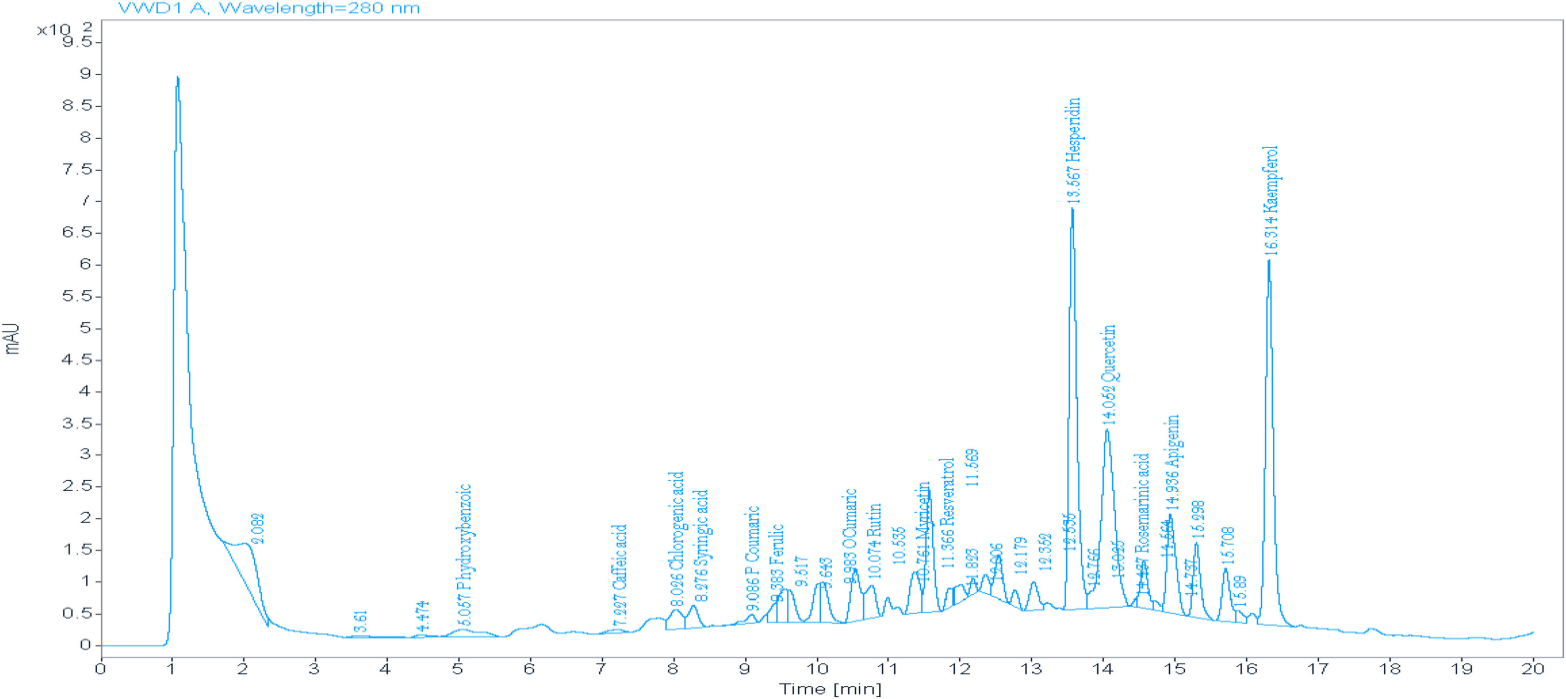
Phenolic components of Hesawi lemon juice.

The HPLC method of LJ detected numerous phytochemical constituents that could be identified. The main peaks at retention times (RT) were Hesperidin, Quercetin, Rosmarinic acid, Apigenin, and Kaempferol (Figure 1). The highest amounts recorded were (135.74, 121.50, and 112.97 mg/kg) for quercetin, hesperidin, and kaempferol, respectively (Table 2).

**TABLE 2:** Phenolic components of lemon juice.

Name	Expected retention time	Retention time (min)	Area	Amount (mg/kg)
p-Hydroxybenzoic acid	4.9	5.057	338.1004	6.71
Caffeic	7.4	7.227	93.0275	1.91
Chlorogenic acid	8.0	8.026	386.6208	14.34
Syringic acid	8.3	8.276	345.5814	5.092
p-Coumaric acid	9.1	9.086	118.5769	0.14
Ferulic	9.3	9.383	174.6429	5.82
o-Coumaric acid	10.2	9.983	426.0624	7.46
Rutin	10.0	10.074	470.3092	11.62
Myricetin	11.1	10.761	507.3779	20.08
Resveratrol	11.4	11.366	608.1003	37.37
Hesperidin	13.3	13.567	4,778.8945	121.50
Quercetin	13.9	14.052	3,668.0356	135.74
Rosmarinic acid	14.4	14.437	45.5406	2.92
Apigenin	15.1	14..936	1,331.7810	37.42
Kaempferol	16.9	16.314	4,329.3950	112.97

### 3.2 Organic acid components of lemon juice

**FIGURE 2:**
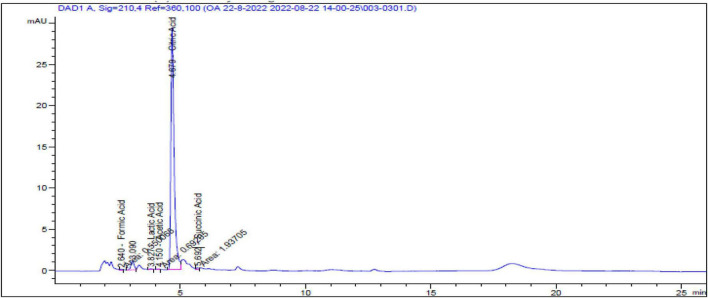
Organic acids component of Hesawi lemon juice.

The highest peak was recorded for citric acid (an acidic antioxidant) with a highest concentration of 38,351.59 μg/mL (Figure 2). The other list of the organic acid components is presented in Table 3.

**TABLE 3:** Organic acids component of lemon juice.

Sample		
Name	Area	Conc. (μ g/mL)
Formic acid	0.55	97.02
Lactic acid	0.57	291.47
Acetic acid	0.69	318.02
Citric acid	262.42	38,351.59
Succinic acid	1.94	1,400.69
Propionic acid	0.00	0.00
Butyric Acid	0.00	0.00

### 3.3 Antioxidant activity and vitamin C content of lemon juice

The results of DPPH in different percentages (2, 5, and 10%) of LJ revealed a positive relationship between the increase in concentration and the percentage of DPPH radical scavenging activity for LJ tested. However, LJ exhibits marked DPPH radical-scavenging properties (Table 4). Concerning the vitamin C content of LJ, a moderate value (36.7 ± 2.4 mg/100 mL).

**TABLE 4:** Antioxidant activity (DPPH)* and vitamin C content of Hesawi lemon juice (LJ).

Test item identifier	DPPH radical scavenging activity %			Vitamin C content (mg/100 mL)
Lemon juice	**2%**	**5%**	**10%**	36.7 ± 2.4
	35.20	61.30	80.24	

*2,2-Diphenyl-1-picrylhydrazyl (DPPH) expresses the percentage inhibition of the DPPH radical.

### 3.4 Body weight change

**FIGURE 3:**
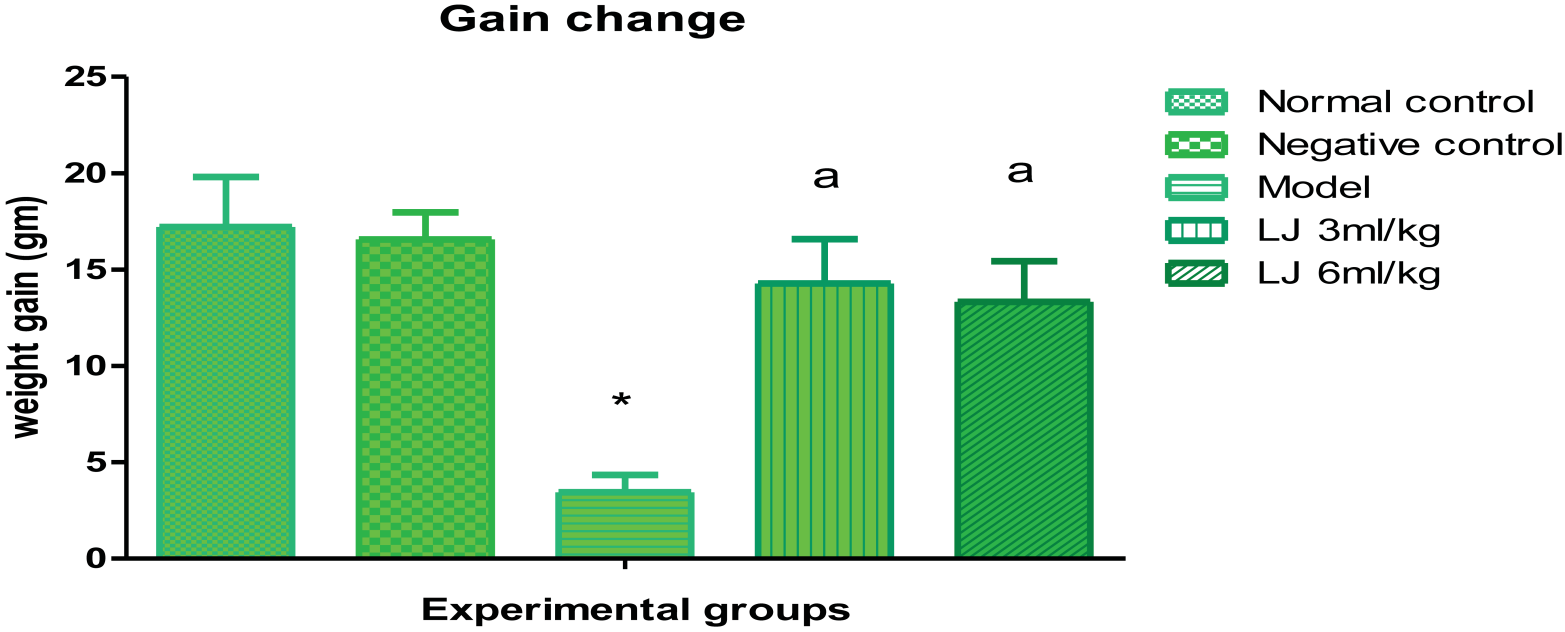
Body weight gain changes of the experimental groups within 1 week. Normal control: no supplementation, no surgical operation; Negative control: no supplementation, with surgical operation by saline; Positive control (Model): no supplementation, with surgical operation by 5% glycolic acid; LJ: lemon juice. (*) is significantly different from the control negative group at *P* < 0.05. (a) is significantly different from the model group at *P* < 0.05.

Body weight gain did not differ significantly between the normal and negative control groups, indicating that the surgical procedure itself had no effect on this parameter. Meanwhile, there was a significant reduction in body weight gain (*P* < 0.05) in the positive control that instilled 5% glycolic acid without LJ supplementation compared to the negative control (Figure 3). At the same time, the groups that received LJ recorded a significant (*P* < 0.05) improvement in body gain compared to the control positive, which nearly returned to the value of the control negative. No mortality was observed among the rats throughout the study period. However, a decrease in activity was observed in the positive control (model) group, which received 5% glycolic acid without supplementation.

### 3.5 Leucogram (total leucocyte count (TLC), differential leucocyte count, neutrophils/lymphocyte (N/L) ratio) and spleen cellular viability %

There was a significant increase in the total leukocyte count (TLC; *P* < 0.01) and neutrophil percentage (*P* < 0.05) in the positive control (model) group, which received intratracheal instillation of 5% glycolic acid, compared to the negative control group (Table 5). Conversely, the group that received a high dose of LJ showed a significant decrease in these parameters, with values returning to levels nearly equivalent to those of the negative control group. The control positive group showed a significant (*P* < 0.05) decrease in lymphocytes (L), and a significant (*P* < 0.05) increase in eosinophils compared with the negative control; however, it became likely to be a negative group in rats that received low and high doses of LJ. The elevated neutrophil-to-lymphocyte (N/L) ratio observed in the positive control group was normalized in rats receiving either dose of LJ. Monocyte percentages and spleen cellular viability were unaffected by LJ supplementation across all experimental groups.

**TABLE 5:** Effect of lemon juice (LJ) on total leucocyte count (TLC), differential leucocyte count, and spleen cellular viability %.

Groups	Normal control	Negative control	Positive control (Model)	Model + LJ (Low dose)	Model + LJ (High dose)	*P*-value
TLC (103/mm^3^)	6.74 ± 0.47	5.43 ± 0.11	10.32 ± 1.42**	6.43 ± 1.90	5.66 ± 1.02^a^	0.0327
Neutrophil (N)%	60.85 ± 4.36	61.53 ± 2.58	75.94 ± 1.76*	65.33 ± 1.70	60.11 ± 1.70^a^	0.0143
Lymphocyte (L)%	30.14 ± 2.75	29.78 ± 1.04	24.20 ± 0.33*	31.51 ± 0.93^a^	31.61 ± 0.74^a^	0.0284
N/L ratio	2.01 ± 0.37	2.36 ± 0.28	3.28 ± 0.13*	2.19 ± 0.11	1.99 ± 0.26^a^	0.0426
Eosinophils%	3.14 ± 0.46	2.67 ± 0.16	5.53 ± 0.11*	2.74 ± 0.21^a^	2.08 ± 0.22^a^	0.0428
Monocytes%	6.12 ± 1.57	6.75 ± 2.44	6.06 ± 3.02	6.12 ± 3.15	6.33 ± 2.42	0.2430
Spleen cellular viability %	93.86 ± 4.33	95.12 ± 2.65	92.49 ± 1.65	91.17 ± 3.39	91.17 ± 3.39	0.6578

Mean ± Standard error (SE). The means having marks (*, **) in the same row are significantly different from the negative control group at *P* < 0.05 and *P* < 0.01, respectively. The means having mark (^a^) are significantly different from the control positive group (Model) at *P* < 0.05. N/L, Neutrophils /Lymphocytes.

### 3.6 Phagocytic activity (phagocytic index, phagocytosis)

The phagocytic index was measured in vitro on days 1 and 7 post-instillation in the glycolic acid-exposed groups, which had received different doses of LJ (Table 6). The phagocytic index increased significantly (*P* < 0.05) in the control-positive group on day 1 after the operation compared with the control-negative group. The groups that received LJ exerted a non-significant increase in phagocytic index. On the contrary, on day 7, the phagocytic index was significantly reduced (*P* < 0.05) in the control positive group that was treated with glycolic acid without LJ supplementation compared to the control negative group; meanwhile, it was significantly elevated (*P* < 0.05) in the group that received a high dose of LJ relative to the control positive group.

**TABLE 6:** Effect of lemon juice (LJ) on the phagocytic index in rats on day 1 and day 7 post intratracheal instillation of glycolic acid.

Sampling day post-operation	Groups	logOD1	logOD2	K = logOD1-logOD2 / t2 – t1 (Phagocytic index)
Day 1	Normal group	1.46 ± 0.46	1.44 ± 0.53	0.0041 ± 0.0001
	Control negative	1.48 ± 0.33	1.42 ± 0.44	0.0039 ± 0.0001
	Control positive (Model)	1.82 ± 0.13	1.58 ± 0.28	0.0173 ± 0.0.009^a^
	Model + LJ (Low dose)	1.43 ± 0.26	1.35 ± 0.29	0.0086 ± 0.0.002
	Model + LJ (High dose)	1.77 ± 0.51	1.62 ± 0.43	0.0070 ± 0.0.001
Day 7	Normal control	1.65 ± 0.47	1.44 ± 0.37	0.0081 ± 0.0.011
	Control negative	1.63 ± 0.33	1.47 ± 0.31	0.0082 ± 0.0.011
	Control positive (Model)	1.72 ± 0.29	1.54 ± 0.09	0.0043 ± 0.0.001*
	Model + LJ (Low dose)	1.69 ± 0.11	1.43 ± 0.26	0.0081 ± 0.0.008
	Model + LJ (High dose)	1.87 ± 0.36	1.44 ± 0.51	0.0105 ± 0.0.002^a^

Mean ± Standard error (SE). The means having a mark (*) in the same column are significantly different from the negative control group at *P* < 0.05. The means having mark (^a^) are significantly different from the control positive group (Model) at *P* < 0.05. OD, optical density; t, time.

### 3.7 Glucose consumption with proliferating lymphocytes

Lymphocytes from the positive control (model) group, when stimulated with LPS, showed a significant (*P* < 0.05) reduction in glucose consumption compared to those from the negative control healthy group. Rats treated with LJ showed a significant (*P* < 0.05) and (*P* < 0.01) improvement in glucose consumption with lymphocytes (12.62 ± 3.61 and 17.72 ± 3.67 mg/dL) for low and high doses, respectively. On the other hand, glucose consumption by lymphocytes stimulated by PHA-P showed a significant improvement (*P* < 0.05) in the group that received a high dose of LJ compared to the control positive health group (Table 7).

**TABLE 7:** Effect of lemon juice (LJ) on glucose consumed with proliferating lymphocytes in the incubation medium (mg/dL) stimulated by either LPS or PHA-P.

Mitogen	Groups	Normal control	Negative control	Positive Control (Model)	Model + LJ (Low dose)	Model + LJ (High dose)
LPS (10 μg /100 μL)	Media	43.74 ± 4.31	43.16 ± 2.63	42.74 ± 3.11	48.23 ± 4.02	45.72 ± 2.87
	Media + cells	41.85 ± 2.84	42.43 ± 1.63	40.41 ± 3.85	43.66 ± 1.73	44.63 ± 2.80
	Media + cells + LPS	31.37 ± 2.73	30.62 ± 1.77	33.83 ± 2.09	29.61 ± 2.94	26.92 ± 1.92
	Net glucose consumption	14.11 ± 2.20	13.69 ± 2.20	7.53 ± 1.96*	12.62 ± 3.61^a^	17.72 ± 3.67^b^
PHA-P (5 g/mL)	Media	43.79 ± 4.82	43.18 ± 3.74	45.62 ± 4.90	43.76 ± 2.11	46.81 ± 3.01
	Media + cells	40.43 ± 3.01	40.43 ± 2.94	40.55 ± 2.32	39.81 ± 3.00	40.81 ± 2.04
	Media + cells + PHA-P	30.62 ± 1.91	31.25 ± 1.77	30.37 ± 1.55	29.72 ± 3.30	22.43 ± 1.42
	Net glucose consumption	10.31 ± 2.63	9.76 ± 2.55	11.35 ± 2.29	10.45 ± 2.11	17.53 ± 1.02^a^

Mean ± Standard error (SE). The means having a mark (*) in the same row are significantly different from the control negative group at *P* < 0.05. The means having marks (^a,^ ^b^) are significantly different from the control positive group (Model) at *P* < 0.05 and *P* < 0.01, respectively. LPS, Lipopolysaccharide; PHA-P, Phytohemagglutinin-P.

### 3.8 Lung tissue antioxidant enzyme activities

The antioxidant enzyme activities determined in lung tissue were GSH-Px, SOD, and CAT. A model group showed a significant reduction (*P* < 0.05) in the activity of the three antioxidant enzymes tested (GSH-Px, SOD, and CAT) compared with the negative control. Rats treated with LJ at both doses showed a significant (*P* < 0.05) elevation in GSH-Px and SOD activities compared to the positive control group (Table 8). However, the high dose of LJ significantly increased CAT activity (*P* < 0.05) compared to the positive control; this effect was not observed in rats receiving the low dose.

**TABLE 8:** Effect of lemon juice (LJ) on lung tissue antioxidant enzyme activities.

Groups	Enzymes activity		
	GSH-Px (mM/mg protein)	SOD (U/mg protein)	CAT (U/mg protein)
Normal control	363.75 ± 11.96	48.65 ± 3.81	20.74 ± 2.72
Negative control	357.23 ± 13.43	46.24 ± 3.08	20.24 ± 2.67
Positive control (Model)	301.13 ± 6.89*	37.44 ± 2.73*	14.21 ± 3.04*
Model + LJ (Low dose)	433.10 ± 11.88^a^	50.24 ± 3.53^a^	17.33 ± 2.81
Model + LJ (High dose)	429.54 ± 10.61^a^	55.82 ± 2.95^a^	24.23 ± 1.43^a^

Mean ± Standard error (SE). The means having a mark (*) in the same column are significantly different from the control negative group at *P* < 0.05. The means having mark (a) are significantly different from the control positive group (Model) at *P* < 0.05. GSH-Px, Glutathione peroxidase; SOD, Superoxide dismutase; CAT, Catalase.

### 3.9 Circulating inflammatory cytokines, TNF-α, IL-6, and CRP

There was a significant elevation (*P* < 0.05) in TNF-α and CRP (15.11 ± 1.34 and 5.90 ± 1.09 ng/mL), respectively, in the model group compared to the negative control. In contrast, this elevation was significant at (*P* < 0.01) for IL-6 (12.09 ± 2.44 pg/mL) in the same group (Table 9). A high dose of LJ supplementation normalized the three parameters, TNF-α, IL-6, and CRP, to be near the values of the negative control, where it recorded a significant reduction in IL-6 and CRP at (*P* < 0.01) and at (*P* < 0.05) for TNF-α compared to the model group. The low dose of LJ produced a significant reduction (*P* < 0.05) in IL-6 level only (8.89 ± 0.54 pg/mL) compared with the model group.

**TABLE 9:** Effect of lemon juice (LJ) on circulating pro-inflammatory cytokines, TNF-α, IL-6, and CRP.

Groups	Inflammatory cytokines		
	TNF-α (pg/mL)	IL-6 (pg/mL)	CRP (ng/mL)
Normal control	10.22 ± 1.87	7.58 ± 1.42	2.45 ± 0.73
Negative control	9.45 ± 1.11	7.67 ± 0.85	2.74 ± 0.22
Positive control (Model)	15.11 ± 1.34*	12.09 ± 2.44**	5.90 ± 1.09*
Model + LJ (Low dose)	13.63 ± 2.58	8.89 ± 0.54^a^	3.11 ± 1.01
Model + LJ (High dose)	9.98 ± 2.09^a^	6.67 ± 1.66^b^	2.53 ± 0.65^b^
*P*-value	*P* < 0.007	*P* < 0.0320	*P* < 0.0002

Mean ± Standard error (SE). The means having marks (*, **) in the same column are significantly different from the control negative group at *P* < 0.05 and *P* < 0.01, respectively. The means having marks (^a,^ ^b^) are significantly different from the control positive group (Model) at *P* < 0.05 and *P* < 0.01, respectively. TNF-α, Tumor necrosis factor-α; IL-6, Interleukin-6; CRP, C-reactive protein.

### 3.10 Histopathological examination

**FIGURE 4:**
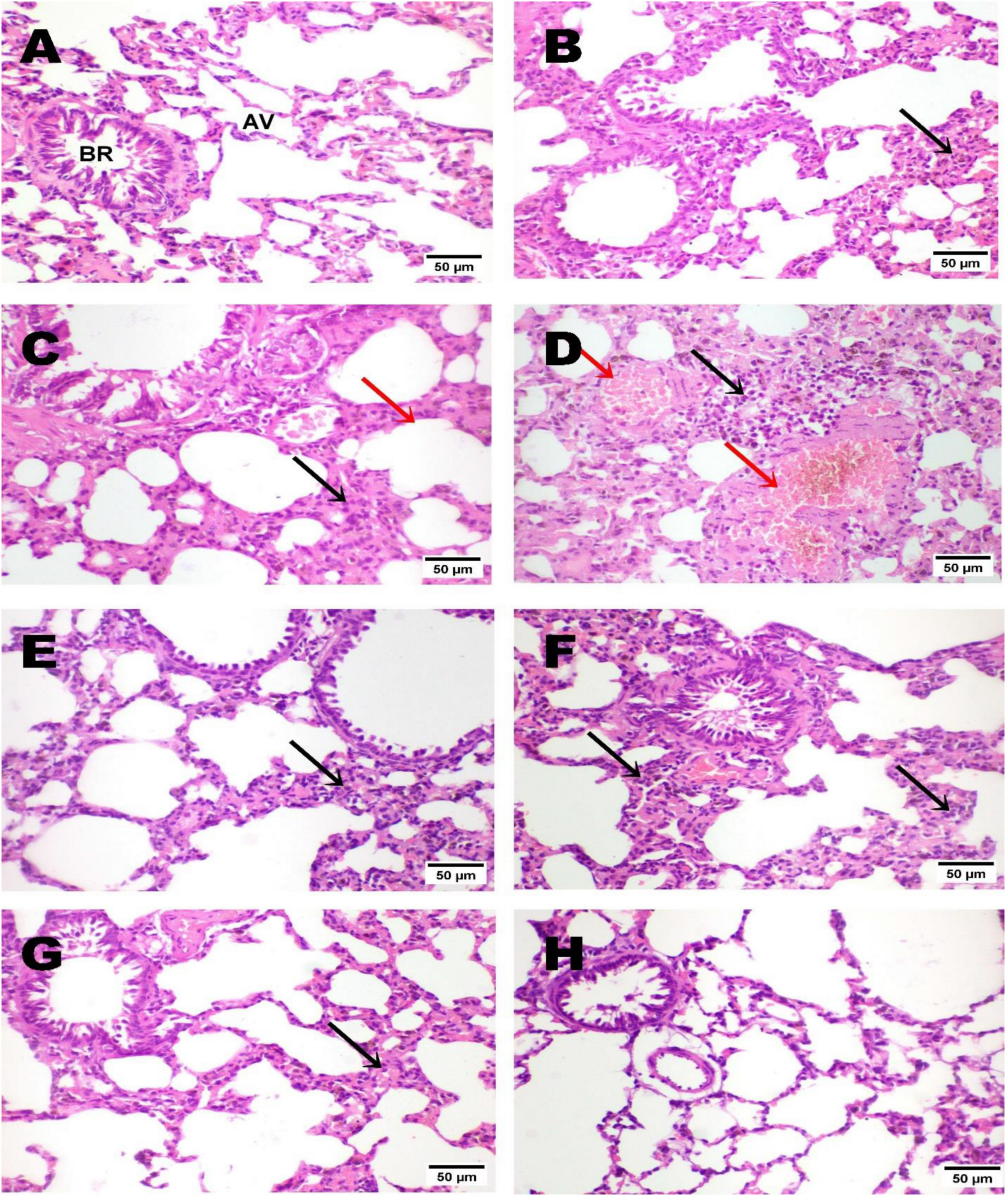
Photomicrograph of lung tissues, note the normal histological architecture of the normal group **(A)**. The negative control showed a slight thickening of interstitial lung tissue **(B)**. Lung tissue from the model group showed marked interstitial septal thickening with inflammatory cells (interstitial pneumonia; black arrow) and giant alveoli (red arrow) **(C)**, along with congestion of pulmonary blood vessels (red arrows) and perivasculitis (black arrow) **(D)**. The lung tissue of the LJ treatment group at a low dose (3 mL/kg) showed a slight thickening of interstitial septa with inflammatory cells (black arrow) **(E)**, with a slight thickening of interstitial tissue (black arrow) **(F)**. The lung tissue of the LJ treatment group at a high dose (6 mL/kg) showed slight thickening of interstitial tissue (black arrow) **(G)**; most sections showed no histopathological alterations **(H)**. Bronchiole (BR); Alveoli (AV); Lemon juice (LJ), (H&E, x200, scale bar, 50 μm).

Microscopic examination of lung tissues from the normal control group revealed a normal histological architecture, including typical bronchioles (Figure 4A). The negative control group exhibited a slight increase in interstitial lung tissue thickness (Figure 4B). In contrast, the lungs of the model group exhibited marked thickening of the interstitial septa, accompanied by inflammatory cell infiltration (interstitial pneumonia) and the formation of giant alveoli (Figure 4C). Along with congestion of pulmonary blood vessels and perivasculitis (Figure 4D). Meanwhile, lung tissue from the low-dose LJ group (3 mL/kg) exhibited slight interstitial septal thickening associated with inflammatory cell infiltration (Figures 4E,F). Although a few lung tissue sections from the high-dose LJ group (6 mL/kg) showed mild interstitial thickening (Figure 4G), most sections showed no histopathological alterations (Figure 4H).

**FIGURE 5:**
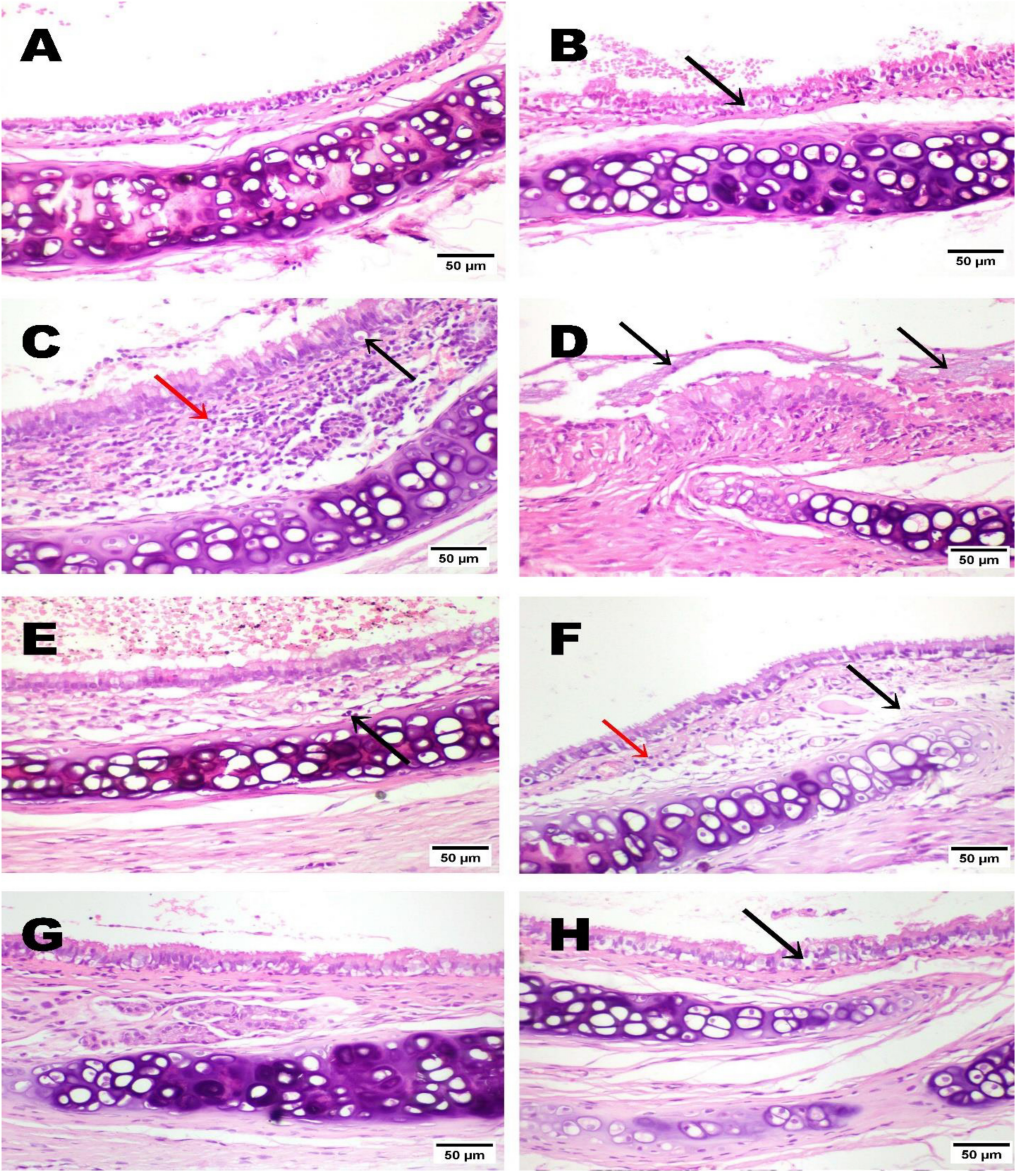
Photomicrograph of tracheal tissue of the normal control showed normal histological architecture **(A)**. The negative control showed a few vacuolations of the tracheal epithelium (arrow) **(B)**. The tracheal tissue of the model group showed apoptosis of mucosal epithelium (black arrow), massive mononuclear cell infiltration in the submucosa (red arrow) **(C)**, and accumulation of bluish mucous exudate in the tracheal lumen (arrow) **(D)**. The tracheal tissue of LJ treatment group at a low dose (3 mL/kg) showed few mononuclear cell infiltration in the submucosa (arrow) **(E)** with slight submucosal edema (black arrow) associated with few mononuclear cell infiltrations in the submucosa (red arrow) **(F)** The tracheal tissue of LJ treatment group at a high dose (6 mL/kg) showed no histopathological lesions **(G)**, with few vacuolations of the tracheal epithelium (arrow) **(H)**. Lemon juice (LJ), (H&E, x200, scale bar 50 μm).

The photomicrograph of tracheal tissue showed normal histological architecture of the normal group (Figure 5A), and the negative control showed a few vacuolations of the tracheal epithelium (Figure 5B). Meanwhile, the tracheal tissues of the positive control (model) exhibited remarkable histopathological alterations. These were characterized by apoptosis of the mucosal epithelium, massive mononuclear cell infiltration in the submucosa (Figure 5C), and accumulation of bluish mucous exudate in the tracheal lumen (Figure 5D). The tracheal tissue of the low-dose LJ group (3 mL/kg) showed slight submucosal edema accompanied by a few mononuclear cell infiltrations (Figures 5E,F). The tracheal tissue of the high-dose LJ treatment group (6 mL/kg) showed no significant histopathological lesions (Figure 5G) aside from some epithelial vacuolation (Figure 5H).

## 4 Discussion

Oxidation, inflammation, and immune response form a complex, interconnected network within various tissues of the body. Oxidative stress is often accompanied by inflammation, which negatively impacts the immune system, and excessive inflammation can harm the body tissues. Reducing oxidative stress is a way to control inflammation and improve immunocompetence. Nutrition is one aspect that influences antioxidants and anti-inflammatory components, which affect the immune response (23, 37). The phytochemical components of plants play crucial roles in regulating oxidative and inflammatory stress, supporting innate and acquired immunity of cellular components (38). The overall results of the current study revealed that the phytochemical and antioxidant capacity of LJ showed that hesperidin is the most abundant flavanone compound. In contrast, the highest flavanols were quercetin and kaempferol, which agrees with many studies (12, 39). Among the bioactive organic acids in LJ, citric acid was found at the highest concentration, followed by succinic acid. DPPH assay indicated that LJ had a high radical scavenging activity percentage, particularly with increasing concentrations. These findings coincide with many researchers who reported that lemon has antioxidant and anti-inflammatory effects (11, 40). The high content of flavonoid compounds, the inhibition of DPPH free radicals, and an elevation in the radical scavenger activity of lemon (39). The vitamin C content of LJ, which was 36.7 mg/100 mL, is considered a moderate value comparable to the range of (45–55) mg/100 mL suggested previously (41, 42) who determined vitamin C using the same method used in the present study. Nevertheless, the obtained vitamin C value is nearly the same as the vitamin C content previously published (43). The moderate amount of vitamin C may be attributed to the labile nature of ascorbic acid, which is affected by environmental conditions (44).

The model group that received intratracheal instillation of glycolic acid without LJ showed reduced body weight gain. This is likely due to lung irritation caused by glycolic acid, which impairs pulmonary function. A decrease in lung function is associated with a reduction in ventilation and oxygen intake and is reported to affect body functions, which consequently affect body weight negatively (45). LJ supplementation improved body weight gain, suggesting a corresponding enhancement in alveolar function mediated by its bioactive constituents. Hesperidin, as a bioactive content of lemon juice, decreases endothelial inflammation in patients with myocardial infarction (46). In addition, citrus juice contains vitamin C, which modulates and protects the endothelial cells; it represents a remedy to save ill patients (47).

The significant adverse alterations in TLC, neutrophil, and lymphocyte counts recorded in the positive control (model) group indicate an inflammatory response induced by intratracheal instillation of glycolic acid (48). A high dose of LJ normalizing TLC and neutrophil may be attributed to the hesperidin, quercetin, and vitamin C content. Previous work indicated that leukocytes were modified by 292 mg of hesperidin per day for 4 weeks (49). Moreover, the quercetin content of LJ may reduce the release of leukocytes, particularly macrophages, consequently alleviating lung inflammation (50). For patients with recurrent infections and impaired leukocyte function, vitamin C has a restorative effect (51). The significant increase in lymphocyte percentage, decrease in neutrophil percentage, and reduction in N/L ratio observed in groups receiving LJ, compared to the model group, indicate beneficial effects on the immune system (52). Healthy adults with vitamin C deficiency had 50% lower levels of vitamin C in their mononuclear cells. This suggests that lymphocyte function may be dependent on vitamin C levels (53). Vitamin C has a role in the differentiation and maturation of immature T cells (54). According to the current findings, the various experimental treatments did not affect the percentage of monocytes and spleen cellular viability.

The significant increase in the phagocytic index in the model group on day 1 post-operation may be attributed to heightened phagocytic activity, an early and expected component of the acute inflammatory response to glycolic acid instillation. This acute phase is referred to as an inflammatory storm (55), which is harmful to the host and may cause multiorgan damage, lethality, and life-threatening systemic inflammatory syndromes involving elevated levels of circulating cytokines and immune cell hyperactivation. This initial immune activation was followed by a significant decline in the phagocytic index by day 7 in the same group, suggesting impairment of this key immune function as inflammation progressed.

The groups treated with LJ, particularly at the high dose, exhibited a stable or improved phagocytic index by day 7. This suggests that LJ supplementation helped sustain effective phagocytic function, likely by mitigating oxidative stress and inflammation, an effect supported by the corresponding reductions in inflammatory cytokines (TNF-α, IL-6) and CRP, and the improvement in lung antioxidant enzyme activities observed in these groups. The slight increase in the phagocytic index in groups treated with LJ indicated a soothing effect on lung irritation induced by glycolic acid, attributable to its bioactive constituents. A patient who consumes vitamin C reduces the length and intensity of colds (56).

On the other hand, on day 7 post-operation, the phagocytic index was significantly reduced in the model (positive control) group. This finding suggests a depletion of functional immune capacity, which may be attributed to the exhaustion of key antioxidants, such as vitamin C, during sustained inflammation. This is consistent with clinical reports showing that patients with acute respiratory infections exhibit depleted leukocyte vitamin C levels, and that supplementation can improve outcomes (57). Therefore, the superior restoration of the phagocytic index by the high dose of LJ likely stems from its provision of vitamin C and other bioactives at levels sufficient to replete these diminished antioxidant reserves and support immune cell function. The link between leukocyte, vitamin C depletion, and clinical vitamin C deficiency in pneumonia patients is crucial; vitamin C plays a role in immune cell function and redox balance (58). Vitamin C is highly concentrated in leukocytes, where it serves as a primary antioxidant and cofactor for numerous enzymes. Its depletion within these cells has direct consequences for their function and, by extension, for organ-level pathology and clinical outcomes (59).

The significant elevation of the phagocytic index in the group that received a high dose of LJ on day 7 indicates enhanced immune function alongside suppressed pulmonary inflammation. The anti-inflammatory and immunostimulatory effects are attributed to the synergistic action of bioactive compounds in LJ, such as hesperidin, quercetin, and vitamin C. These components are known to upregulate phagocytic activity and improve the function of immune cells, including neutrophils and natural killer cells (6, 60). Notably, the high dose of LJ demonstrated superior anti-inflammatory efficacy compared to the low dose. This dose-dependent effect may be partly attributed to the pharmacokinetics of vitamin C during inflammation. During oxidative stress and inflammation, vitamin C, a potent antioxidant, is rapidly consumed, leading to depleted plasma and tissue levels (61). The moderate vitamin C content of LJ detected in the present study (36.7 mg/100 mL) in the low-dose group may have been insufficient to compensate for this depletion during glycolic acid-induced pulmonary inflammation. In contrast, the high-dose LJ provided a proportionally greater amount of vitamin C and other antioxidants, potentially reaching a threshold necessary to mitigate oxidative stress, reduce inflammatory cytokine production, and support immune cell function. This aligns with clinical observations that patients with acute respiratory illnesses, such as pneumonia and tuberculosis, often exhibit significantly lower plasma vitamin C concentrations (61), underscoring the heightened demand for this vitamin during inflammatory lung conditions.

The significant increase in glucose consumption by lymphocytes stimulated with LPS or PHA-P in the high-dose LJ group may be attributed to bioactive compounds such as hesperidin, quercetin, and kaempferol, which are known to enhance immunity and regulate inflammation (6). To prevent the negative effects of oxidative stress, immune cells like phagocytes and lymphocytes, which utilize reactive oxygen species (ROS) for many of their functions, require adequate quantities of intracellular antioxidants (62). Hesperidin is a bioactive substance that can reduce oxidative stress, cytotoxicity, and inflammation (63). Hesperidin may enhance immunocompetence and reduce radiation-induced inflammation in mice (64). In acrolein-inhaled mouse models, *Citrus junos* Tanaka and its bioactive compounds reduced lung damage (65). The immune system is supported by a combination of substances found in citrus fruit juices that reduce inflammation and oxidative stress (49). The low dose of LJ significantly improved glucose consumption in LPS-stimulated lymphocytes but not in PHA-P-stimulated ones. This selective activity suggests that the bioactive substances in the low dose are insufficient to produce a broad cell-mediated immune modulatory effect, which may explain its inability to mitigate the pulmonary damage caused by glycolic acid. This indicates that the overall efficacy is dose-dependent.

The significant reduction in the lung activities of GSH-Px, SOD, and CAT caused by intratracheal instillation of glycolic acid indicates inflammation, which, consequently, is followed by oxidative stress and damage to lung tissue (19). The ameliorated effect in the activities of lung GSH-Px and SOD that was recorded in groups that received both doses of LJ, and in CAT activity in the group offered a high dose, might be related to the bioactive polyphenol constituents (particularly quercetin, hesperidin, and kaempferol) and other antioxidant components of lemon juice, like citric acid and vitamin C (6, 49). The citrus peel contains a variety of bioactive and well-known active chemicals that can reduce oxidative stress and inflammation (20). These results align with studies demonstrating that lemon significantly elevates glutathione (GSH) levels and the activities of SOD, CAT, and GSH-Px, and exhibits substantial radical-scavenging activity (20, 66).

The high content of citric acid detected in the present work may have a potential effect as a scavenger of ROS (12). Moreover, the benefits of LJ, particularly at the high dose, may be attributed to its high quercetin content, as detected by HPLC. This suggestion coincides with a recent study, which concluded that quercetin fights oxidative stress and lung inflammation in pulmonary diseases (50). Antioxidant vitamins, including vitamin C, may be useful for the pretreatment of some acute respiratory disorders (66, 67). Furthermore, lemon peel extract has been shown to mitigate fine dust-induced lung injury in mice by reducing inflammatory cells in bronchoalveolar lavage fluid and improving pulmonary antioxidant capacity (20). Similarly, *Citrus junos* Tanaka peel and its bioactive component, naringin, attenuate particulate matter-induced respiratory injury by enhancing pulmonary antioxidant defenses (65). Collectively, these studies showed that the protective effect of citrus derivatives is linked to their anti-inflammatory and antioxidant properties (67, 68). For instance, citrus peel extract demonstrated potent antioxidant effects against particulate matter (PM10)-induced oxidative stress, which triggers apoptosis and inflammatory responses in mouse lungs (20).

To investigate the underlying potential protective mechanism of LJ, the inflammatory cytokines TNF-α and IL-6, as well as CRP, were analyzed. TNF-α is a pro-inflammatory cytokine and is secreted by immunocytes such as macrophages, monocytes, and neutrophils; it responds to inflammation by mediating the induction of neutrophils and eosinophils during airway inflammation (68). Circulating CRP is an indicator of the inflammatory response. It increases following interleukin-6 secretion by macrophages and T cells. Its physiological role is to bind to lysophosphatidylcholine expressed on the surface of dead or dying cells to activate the complement system via C1q (69) The significant increase in the circulating TNF-α, IL-6, and CRP in the model group indicated inflammatory cytokines of the lung tissue due to oxidative stress of glycolic acid instillation, as previously suggested (19). The elevated levels of inflammatory cytokines obtained in the present study in the model group instilled with glycolic acid were considered markers of lung inflammation, as reported previously (5). Glycolic acid, which induces pulmonary oxidative stress, is associated with the release of various cytokines, such as TNF-α, IL-6, and CRP, which can promote lung damage (70). Increased oxidative stress could be involved in cytokines and adipokines dysregulations, including decreased adiponectin transcription and increased levels of leptin, TNF-α, and CRP (71).

The decreased levels of inflammatory cytokines (TNF-α and IL-6) and CRP in the groups treated with LJ, particularly at a high dose, where the reduction was significant, owing to its antioxidant properties that inhibited TNF-α production (72). Administration of hesperidin, one of the main flavonoids in *Citrus limon*, reduced CRP levels in subjects with metabolic syndrome (73). Likewise, hesperidin may enhance immunocompetence and decrease irradiation-induced inflammation in mice through lower concentrations of serum IL-6 and TNF-α (63, 64). Moreover, the anti-inflammatory effect of *Citrus limon* is probably due to the high concentration of D-limonene, a main compound of monoterpenoids present in *Citrus limon* oil (73, 74). The high dose of LJ, which provides a moderate amount of vitamin C, may modulate inflammatory cytokine production, as vitamin C treatment has been shown to reduce microglial cell activation and decrease the synthesis of pro-inflammatory cytokines (TNF-α, IL-6, and IL-1). The overall inflammatory cytokine and CRP results indicate that LJ contains a mixture of components that control oxidative stress and inflammation, thereby enhancing immune function, a finding consistent with the bioactive components identified in this study.

Histopathological examination of lung and trachea specimens in different experimental groups confirmed the results obtained from TLC and differentiation, phagocytic index, glucose consumption by lymphocytes, lung tissue antioxidants, inflammatory cytokines, and CRP. Lung and trachea tissues of the positive control (model) showed inflammatory cells, interstitial pneumonia with the formation of giant alveoli, congestion of pulmonary blood vessels, and perivasculitis induced by intratracheal instillation of glycolic acid (for lung), and remarkable alterations characterized by apoptosis of mucosal epithelium, congestion of submucosal blood vessels, massive mononuclear cell infiltration in the submucosa, with necrosis of the mucosa and accumulation of bluish (for trachea). The findings indicated that glycolic acid is a corrosive and irritative agent on lung tissue (19). Furthermore, LJ administration (Groups 4 and 5) attenuated the inflammation and irritation induced by glycolic acid, an effect that was more pronounced in Group 5, which received the high dose. The lung tissue showed no significant histopathological alterations, aside from a slight thickening of the interstitial tissue. The tracheal tissue also showed no major lesions, although a few sections exhibited epithelial vacuolations. Meanwhile, group 4 (which received a low dose of LJ) showed normal bronchioles and alveoli with a slight thickening of interstitial septa with inflammatory cells (for the lung). Slight submucosal edema with moderate mononuclear cell infiltration in the submucosa (for trachea). Likewise, the histopathological findings in the tracheal tissues mirrored those in the lung specimens. Observations obtained revealed that LJ, in a dose-dependent manner, can ameliorate the inflammatory effect induced by glycolic acid. These findings align with a previous study (5), which concluded that citrus juice by-products ameliorated inflammatory cell infiltration and protected the lungs from particulate-induced damage.

## 5 Conclusion

Finally, according to the present results of the leucogram, phagocytic index, glucose consumption by lymphocytes, antioxidant enzyme activities of lung tissue, inflammatory cytokines, CRP, and histological examination, it is evident that LJ has an ameliorating effect against lung damage induced by surgical intratracheal instillation of glycolic acid. Nevertheless, the LJ benefit effect was dose-dependent, suggesting that the moderate vitamin C content in the low dose of LJ was insufficient to produce the expected mitigative effect, which was only achieved with the high dose. Conclusively, the bioactive contents of LJ control oxidative stress and inflammation, and enhance the immune system, subsequently ameliorating lung tissue irritation induced by surgical intratracheal instillation of glycolic acid in a dose-dependent manner.

## Data Availability

The original contributions presented in the study are included in the article/supplementary material, further inquiries can be directed to the corresponding author.
